# A laboratory simulation to investigate effects of moistures on U distribution among solid phase components in army range soils

**DOI:** 10.1016/j.mex.2022.101678

**Published:** 2022-03-28

**Authors:** Precious Cooper, Lanre Olafuyi, Naira Ibrahim, Joseph Kazery, Steven L. Larson, John H. Ballard, Ahmet Celik, Shaloam Dasari, Saiful M. Islam, Fengxiang X. Han

**Affiliations:** a Department of Chemistry, Physics and Atmospheric Science, Jackson State University, Jackson, MS 39217, USA; b U.S. Army Engineer Research and Development Center, Vicksburg, MS 39180-6199, USA; c Department of Biology, Jackson State University, Jackson, MS 39217, USA

**Keywords:** Yuma soil, Uranium, Distribution, Fractionation, Soil moistures, Biogeochemical conditions, U fractionation, U redistribution

## Abstract

Uranium is a naturally occurring radioactive trace element found in rocks, soils, and coals. U may contaminate groundwater and soil from nuclear power plant operations, spent fuel reprocessing, high-level waste disposal, ore mining and processing, or manufacturing processes. Yuma Proving Ground in Arizona, USA has been used depleted uranium ballistics for 36 years where U has accumulated in this army testing site. The objective of this study is to develop a laboratory scheme on the effects of soil moisture regiments on the distribution and partitioning of U in army range soil among solid phase components to mimic U biogeochemical processes in the field. Three moisture regiments were saturated paste, field capacity, and wetting-drying cycle which covered major scenarios in fields from the wet summer season to the dry winter season. Uranium in soils with different forms of U (UO_2_, UO_3_, uranyl, and schoepite) was fractionated into 8 operationally defined solid components with sequential selective dissolution procedure. The essences of this new development were as following:

• A scheme was developed for investigation of U distribution, partitioning and transformation among solid phase components in army weapon test range soils with various U forms under 3 soil moisture regimes.
• Soil moisture was one of major environmental factors in controlling biogeochemical processes and fates of U in army weapon test site.

## Graphics abstract

**Figure:**
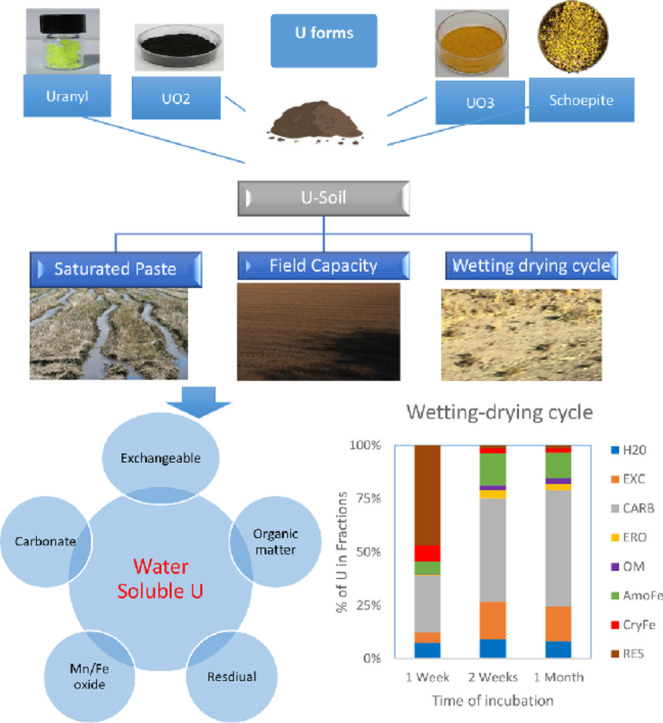

**Specifications table**

**Table:** 

**Subject Area:**	*Environmental Science*
**More specific subject area:**	*Heavy metal pollution control and remediation*
**Method name:**	A laboratory simulation to investigate effects of moistures on U distribution among solid phase components in army range soils
**Name and reference of original method:**	*Kazery, J.A., Proctor, G., Larson, S.L., Ballard, J.H., Knotek-Smith, H. M., Zhang, Q., Celik, A., Dasari, S., Islam, S. M., Tchounwou, P.B., Han, F. X.* (2021). Distribution and Fractionation of Uranium in Weapon Tested Range Soils. American Chemical Society Earth and Space Chemistry 5, 356–364.
**Resource availability:**	*N/A*

## Method details

### Background

Uranium is a naturally occurring heavy metal with radiotoxicity and chemical toxicity and is found in the range of 0.3-11.7 mg kg^−1^ in rocks and soils with an average of 3 mg kg^−1^ [1,2]. Natural uranium is a combination of three radioisotopes which are identified by the mass numbers ^238^U (99.27%), ^235^U (0.72%) and ^234^U (0.0054%) [3]. Depleted uranium (DU) is the by-product of the U enrichment process. DU is used in military applications based on its pyrophoric properties and high density. DU is an effective material for armor-piercing projectiles such as penetrators and for armor-plating tanks. Several hundred tons of DU were used in military conflict over the past forty years and DU is expected to remain as products of corrosion products (U oxides and hydroxides) in battlefields such as Gulf War and army testing sites such as Yuma Proving Ground [4], [5], [6], [7].

The transport of U in soil was affected by various biogeochemical conditions and environmental factors, such as the initial uranium solubility of the corrosion product, pH values of soil, soluble ions present in the soil pore water, mass of organic matter in soil, sizes of soil particles, soil moisture regimes, and soil texture. Under arid conditions, both vertical transport driven by evaporation (upward) and leaching (downward), as well as horizontal transport of U driven by surface runoff in the summer were observed in fields [8,9]. Upward vertical transport of U, as soluble uranyl, was simulated and confirmed under laboratory-controlled conditions which migrates to the surface due to capillary action via evaporation during alternating wetting and drying conditions [8,9]. In the field, the 92.8% of uranium from DU penetrators and fragments remained in the top 5 cm of soil profile and decreased to background concentrations in less than 20 cm of the distance from the source. In locations prone to high amounts of water runoff, U concentrations were reduced significantly after 20 m from the source due to increased inundation. U was also transported throughout the ecosystem via plant uptake and wild animal consumption between trophic levels, but with limited accumulation in edible portions in plants and some animals [8]. Zhang et al. [10] reported that soil moisture regimes played a significant role in DU metal corrosion and migration. They reported that UO_2.8_ and UO_3_ appeared in the DU metallic fragment/soil systems under the saturated soil regime in 4 weeks. UO_3_ was observed in the soil with the field capacity moisture regime in 16 weeks, but not in the air dry soil. DU metal corrosion rates decreased following the trend: Saturated soil > Field capacity soil > Air dry soil.

Heavy metals are transformed and redistributed in soils under various soil moisture. Saturated paste regimes have been shown to increase heavy metals in the carbonate bound fraction and reduce the easily reducible oxide fractions [11], [12], [13]. Saturated regimes increase added heavy metals in the carbonate fraction while field capacity/wetting-drying cycle regimes increase added heavy metals in the easily reducible oxide, organic matter, and iron oxide fractions as indicated by the sequential selective analyses. However, long term saturated paste moisture increased solubility of most native trace elements such as Fe, Mn, Co, and Cu in the arid soil due to reduction of Mn oxides, followed by iron oxides [14,15].

In soil and sediments, heavy metals including U tend to partition among the solid-phase components and solution phases. These included water soluble (Water), clay surfaces (EXC), carbonates (CARB), easily reducible oxide (ERO) such as manganese oxides, organic matter (OM), iron oxides (RO), and residual phase (RES) [14,16].

The main objective of this study is to develop the laboratory scheme to investigate the effects of various soil moisture regimes on the distribution and partitioning of U among solid phase components to mimic the biogeochemical processes in the field. These moisture regiments include saturated paste, field capacity, and wetting-drying cycle which covered major soil moistures in fields from stormy seasons to dry seasons.

## Procedures

1. All persons with this study should first attend the Radiosafety training and got the safety certificate after passing the test.
2. All experiments were conducted in the hood and persons wearied chemical gloves and 3M masks according to OSHA standards.
3. A composite clean arid soil sample was formed from five subsamples taken from fields at 0-15 cm depth in Yuma Proving Ground in Yuma, AZ, USA. Soil was air dried and ground through 2 mm sieve.
4. Uranyl, UO2, UO3 were purchased from United Nuclear Scientific Equipment and Supplies. Schoepite, a corrosion product from DU penetrators was collected from DU contaminated fields in Yuma Proving Ground [6,7].
5. All U chemicals and schoepite samples were ground with agate motors to achieve the uniform fineness.
6. Soil was spiked with 100 mg kg−1 U with the gradual dilution method [17]. A 500 g soil with 100 mg kg−1 U in various forms was mixed into a plastic beaker with a cap (Fig. 1).

**Fig. 1:**
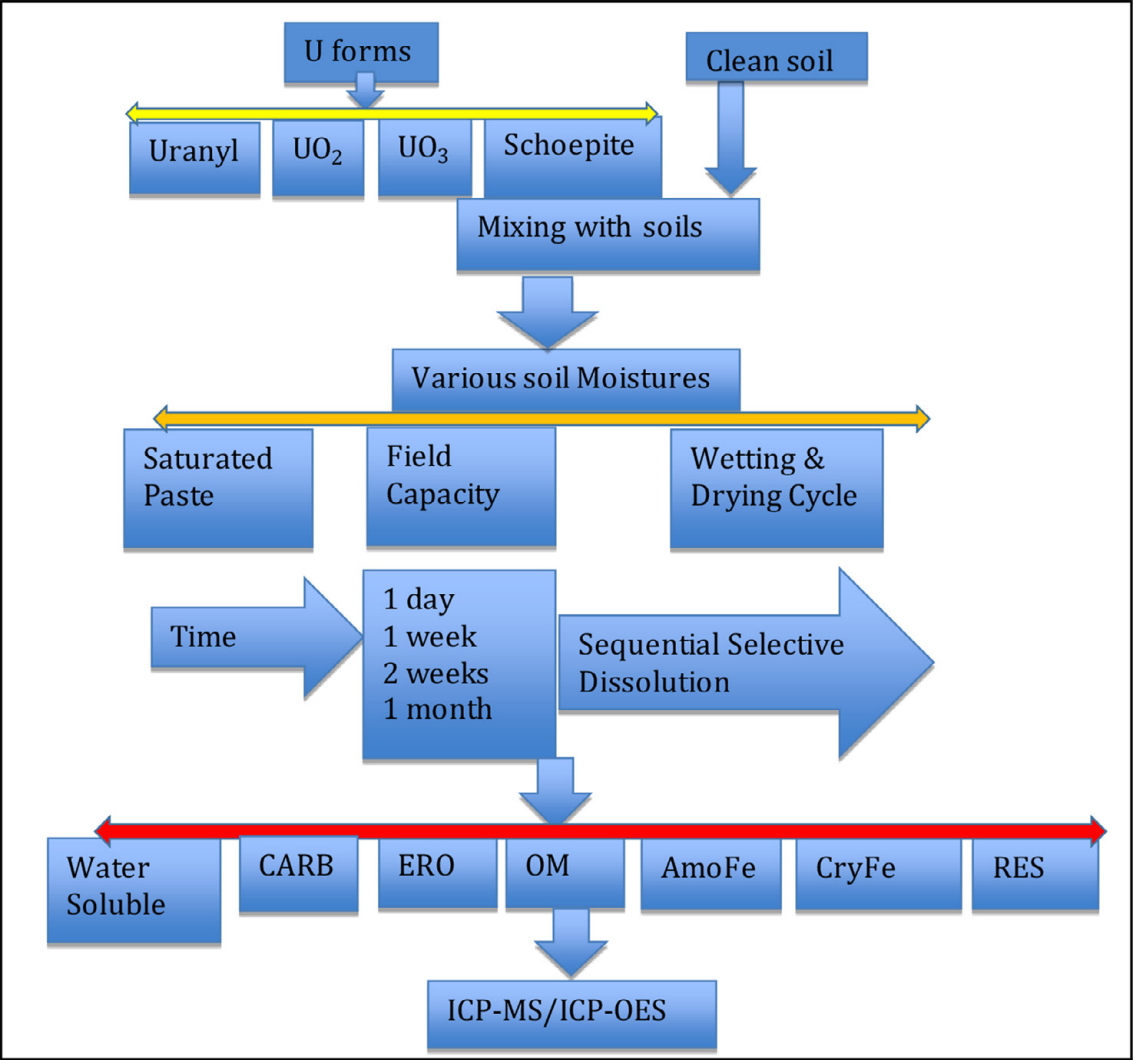
Flow chart of the laboratory scale simulation on effects of soil moisture on U distribution among solid-phase components in soils. Water (H_2_O): the water soluble fraction, EXC: the exchangeable, CARB: the carbonate bound, ERO: the easily reducible oxide bound, OM: the organic matter bound, AmoFe: the amorphous iron oxide bound, CryFe: the crystalline iron oxide bound, RES: the residual fraction, ICP-MS/ICP-OES: inductively coupled plasma- mass spectroscopy/optical emission spectroscopy.
7. Three moisture regimes, saturated paste, field capacity, and wetting-drying cycle regimes were employed in this study. Saturated moisture (25% moisture) was determined [14]. The field capacity was determined by 70% of water saturation. For the wetting-drying cycle the same method was used with field capacity and left open to air dry.
8. The U spiked soils were incubated at the room temperature in three moisture regimes. Each treatment was duplicated.
9. U spiked soil was incubated for 1 day, 1 week, 2 weeks, and 1 months according to the previous studies [11], [12], [13], [14]. Soil moisture was kept constant by adding water to the specific water moisture regime over the period of incubation.
10. At a specific time, a subsoil sample was taken for sequential dissolution analysis as described below. At the same time, a subsample was taken to determine the soil moisture.
11. U in soils was fractionated with sequential selective dissolutions.

The sequential selective dissolution included 8 operationally defined fractions: Water soluble (Water), Exchangeable (EXC), Carbonate (CARB), Easily reducible oxide (ERO), Organic matter (OM), Amorphous Fe oxide (AmoFe), Crystalline Fe oxide (CryFe), and Residual (RES) fractions [7,^14^,16].

11.1. Water soluble U (Water). Approximately 1 g of ground soil was weighed into a 50 mL centrifuge tube. Twenty-five mL of deionized water was added to soil. The mixture was shaken for 2 hours and then centrifuged. The supernatant was collected and filtered through a 0.45 μm filter for determination of U in solution with ICP-MS/ICP-OES. The residual soil was kept for the next step.
11.2. Exchangeable U (EXC). The remainder of soil, after water extraction was added with 25 mL of 1M NH_4_NO_3_ with pH of 7.0 adjusted with ammonium hydroxide and diluted nitric acid. The sample was then agitated for 30 minutes at room temperature then centrifuged. Supernatant was then decanted, filtered, and analyzed as in the previous step, which was also followed in subsequent fractions.
11.3. U bound to carbonate (CARB). The soil residue from the previous extraction was used. Twenty five mL of CH_3_COONa buffer solution at pH 5.00 were added to the soil. The pH of CH_3_COONa buffer solution was adjusted with ammonium hydroxide and diluted nitric acid. The mixture was agitated for 6 hours then centrifuged for separation.
11.4. U bound to easily reducible oxides (ERO). The soil residue from the previous extraction was used by adding 25 mL of 0.01 M HCl and agitated for 30 minutes. The mixture was then centrifuged for separation.
11.5. U bound to organic matter (OM). The soil residue from the previous extraction was used by adding 3 mL of 0.01 M HNO_3_ and 5 mL of 30% H_2_O_2_ and digested in a water bath of 80°C for 2 hours. Then, 2 mL of 30% H_2_O_2_ was added and heated for an additional hour. After heating, 15 mL of 1 M NH_4_NO_3_ was added to the mixture and agitated for 10 minutes. The sample was then centrifuged for separation.
11.6. U bound to amorphous iron oxides (AmoFe). The soil residue from the previous extraction was mixed with 25 mL of 0.2 M oxalate buffer and agitated for 4 hours. The sample was then centrifuged for separation.
11.7. U bound to crystalline iron oxides (CryFe). The previous soil residue was mixed with 25 mL of 0.04 M NH_2_OH•HCl in 25% acetic acid and digested in a water bath at 97-100°C for 3 hours. The mixture was then centrifuged for separation.
11.8. U in the residual fraction (RES). Twenty-five mL of 4 M HNO_3_ was added to the soil residue from the previous step and digested in water bath at 80°C for 16 hours.

12. The supernatants were then diluted and U concentrations in solution was measured with inductively coupled plasma-mass spectrometer (ICP-MS) and inductively coupled plasma-optic emission spectrometer (ICP-OES).

This work provides a comprehensive laboratory simulation scheme for investigating the biogeochemical process of U in soils, such as army weapon-tested range sites as affected by soil moistures (Fig. 1). This study combined the U sources (uranyl, UO_2_, UO_3_, and schoepite) in soils with soil moistures (saturated paste, field capacity, and wetting-drying cycle) through sequential selective dissolution procedures to study redistribution of U among solid phase components. U in soils was divided into the following fractions: water soluble fraction, exchangeable fraction, U bound to easily reducible oxide, U bound to organic matter, U bound to amorphous iron oxide, U bound to crystalline iron oxide, and residual fraction.

**Fig. 2:**
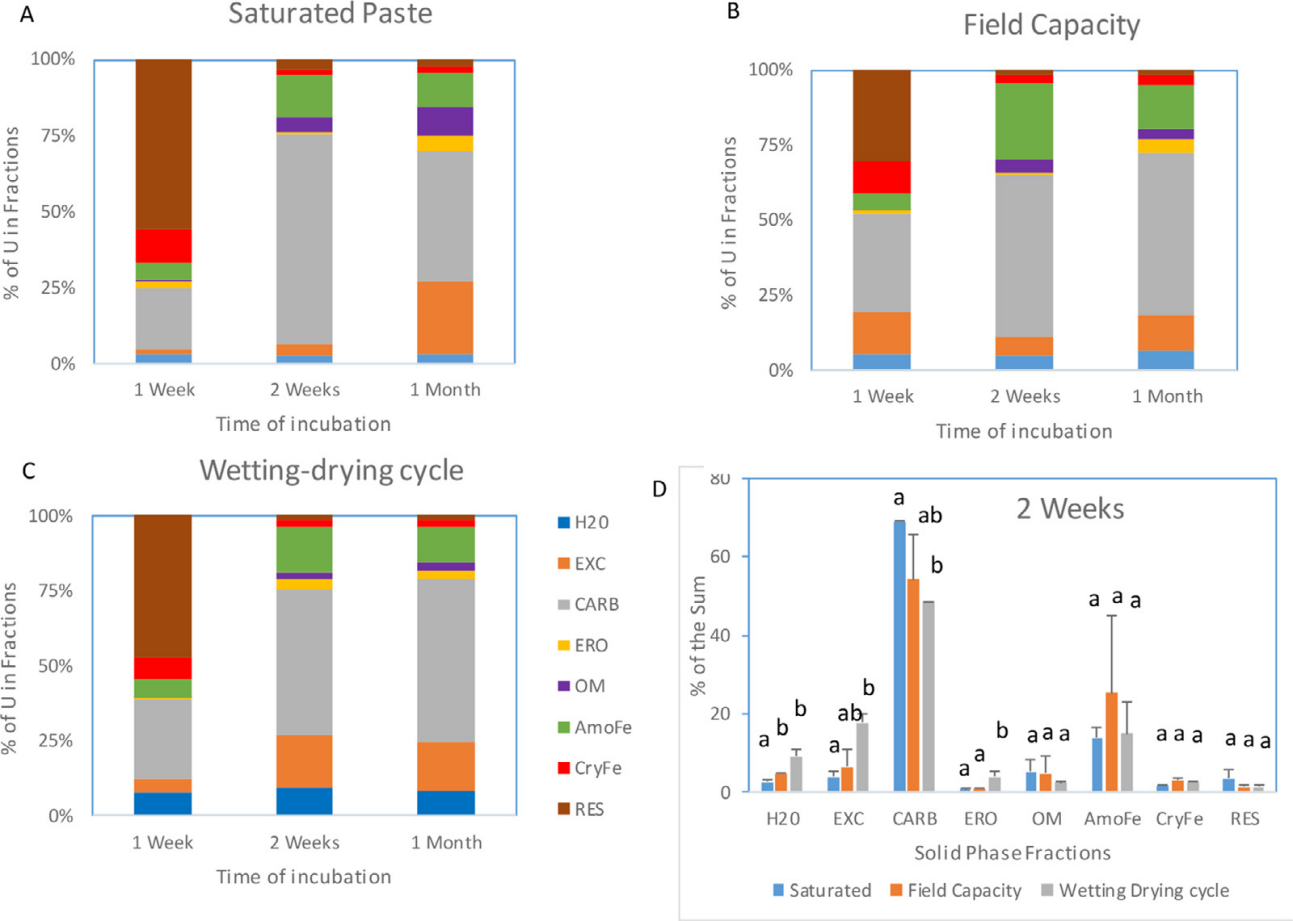
U distribution among solid-phase components in Yuma soils with uranyl at 100 mg/kg under saturated paste (A), field capacity (B) and wetting-drying cycle (C) moistures over 1 week, 2 weeks and 1 month of incubation. D. The comparison of U distribution among three moisture regimes at 2 weeks of incubation. H_2_O: the water soluble fraction, EXC: the exchangeable, CARB: the carbonate bound, ERO: the easily reducible oxide bound, OM: the organic matter bound, AmoFe: the amorphous iron oxide bound, CryFe: the crystalline iron oxide bound, RES: the residual fraction. In Fig. 2D, difference letters in the same fraction indicate the significant difference at *p* = 0.05 probability among three moistures.

**Method validation**. Three soil moistures mimicked all field scenarios from the summer stormy season to the winter dry season, occurring at Yuma Proving Ground [7]. Fig. 2 shows that significant difference in U distribution among solid component fractions as affected by soil moisture, especially in Water soluble (Water), exchangeable (EXC), carbonate (CARB) and easily reducible oxide (ERO) bound fractions (Fig. 2D). Saturated paste resulted in the increase in the CARB fraction (CARB) while in the decreases in Water, EXC and ERO fractions after 2 weeks, compared with Field capacity and Wetting-drying cycle regime. Wetting-drying cycle regime had the increase in Water, EXC, and ERO, but the decrease in the CARB fraction (Fig. 2)

Soil moisture controls redox potential in soils and soil acidity [14], which shifts the forms and species of heavy metal and trace elements including U in soils. The dissolution and transformation of metaschoepite was controlled by water regimes and redox processes in soil system [18]. Soil moisture regimes played a significant role in rates of DU metal corrosion and migration [10]. DU metal corrosion rates decreased from saturated soil to field capacity soil, followed by air dry soil. Therefore the laboratory scheme proposed in this study was not only for studying biogeochemistry of U in range soils, but also for examining biogeochemical processes of other heavy metals and trace elements in soils, sediments, and specific ecosystems.

## Declaration of Competing Interests

The authors declare that they have no known competing financial interests or personal relationships that could have appeared to influence the work reported in this paper.
